# The effect of low dietary inflammatory index score formula on inflammatory, metabolic, and clinical outcomes in critically ill traumatic brain injury patients: A single‐blind randomized controlled pilot study

**DOI:** 10.1002/fsn3.3326

**Published:** 2023-04-19

**Authors:** Sajedeh Jandari, Reza Rezvani, Sajedeh Yousefian, Negin Mosalmanzadeh, Mohammad Bagherniya, Davood Soleimani, Seyedeh Zeynab Mousavian, Nitin Shivappa, James R. Hébert, Ali Jafarzadeh Esfahani, Abass Akhgari, Lida Jarahi, Mohammad Safarian

**Affiliations:** ^1^ Department of Nutrition, Faculty of Medicine Mashhad University of Medical Sciences Mashhad Iran; ^2^ Department of Nutrition Sciences Varastegan Institute for Medical Sciences Mashhad Iran; ^3^ Department of Community Nutrition, School of Nutrition and Food Science Isfahan University of Medical Sciences Isfahan Iran; ^4^ Department of Nutritional Sciences, School of Nutritional Sciences Kermanshah University of Medical Sciences Kermanshah Iran; ^5^ Cancer Prevention and Control Program University of South Carolina Columbia South Carolina USA; ^6^ Department of Epidemiology and Biostatistics, Arnold School of Public Health University of South Carolina Columbia South Carolina USA; ^7^ Connecting Health Innovations LLC Columbia South Carolina USA; ^8^ Targeted Drug Delivery Research Center Pharmaceutical Technology Institute, Mashhad University of Medical Sciences Mashhad Iran; ^9^ Department of Pharmaceutics, School of Pharmacy Mashhad University of Medical Sciences Mashhad Iran

**Keywords:** dietary inflammatory index, enteral nutrition, formula, inflammation, traumatic brain injury

## Abstract

In traumatic brain injury (TBI) patients, a complex cascade of inflammatory responses are frequently observed following trauma. Numerous dietary agents have long been found to have potential in modulating inflammatory responses. This pilot study, designed an enteral formula with low inflammatory properties based on the dietary inflammatory index (DII®) and evaluated its effect on inflammatory and metabolic factors in critically ill TBI patients. This single‐blind randomized controlled pilot study was conducted at the Neurosurgical ICU of Shahid Kamyab Hospital (Mashhad, Iran). A total of 20 TBI patients were randomly assigned to receive either low‐DII score or standard formula at the intensive care unit (ICU). The primary outcomes of the study included clinical status, inflammatory biomarkers, APACHE II, SAPS II, SOFA, and NUTRIC scores. The trial groups did not differ significantly in baseline values. Following 14 days of intervention, there was a statistically significant decrease in the APACHE II, SAPS II, and NUTRIC scores and a significant increase in the GCS score in the low‐DII score formula group compared to the standard formula group. Over 2 weeks, high sensitivity C‐reactive protein (hs‐CRP) values of −2.73 (95% CI: −3.67, −1.79) mg/dL in the low‐DII score formula group versus 0.65 (95% CI: −0.29, 1.58) mg/dL in controls were obtained. Moreover, the length of hospital stay was longer for the standard formula group than for the low‐DII score formula group. The low‐DII score formula improves inflammatory factors (serum hs‐CRP) and metabolic biomarkers (LDL‐c and FBS). Furthermore, clinical outcomes, including the length of hospital stay and disease severity, appear to be enhanced.

## Graphical abstract

This pilot study aimed to assess the effects of this low‐DII score enteral formula on metabolic, inflammatory factors, and clinical outcomes of patients admitted to intensive care units (ICU) with traumatic brain injury (TBI).

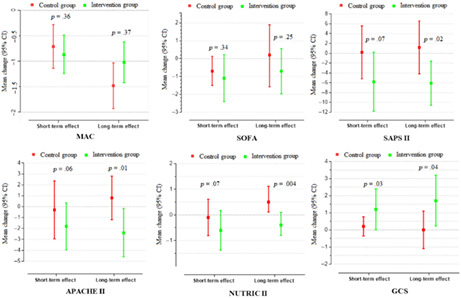

## 1 INTRODUCTION

Traumatic brain injury (TBI) remains a challenging issue in critical care medicine due to its contribution to the majority of trauma‐related deaths (Baker et al., 1980; Rusnak, 2013; Shackford et al., 1993). TBI refers to noncongenital brain injury, initiated with focal or diffuse lesions at the time of the primary injury, and possibly prolonged and exacerbated by a set of complex systemic and local events that results in secondary brain injury (Prasetyo, 2020). The secondary injury is found to be promoted by several diverse etiologies, including metabolic changes, inflammation, ischemia, edema, and excitotoxicity (Algattas & Huang, 2014).

In the last two decades, investigators have made great progress in understanding the main mechanistic pathways linking inflammation to secondary brain injury after trauma (Lenzlinger et al., 2001). Post‐traumatic neuroinflammation, the neurogenic inflammation following TBI, is primarily developed by several extracellular and intracellular signaling pathways and is found to be a key contributor to secondary injuries (Prasetyo, 2020; Wofford et al., 2019). TBI also initiates systemic inflammatory response syndrome (SIRS), which is found to be a potential factor in raising the risk of nosocomial infection or multiple organ dysfunction (MOD) (Simon et al., 2017). SIRS score potentially predicts the length of hospital stay (LOS) and patients' mortality (Jacome & Tatum, 2018). In the intensive care unit (ICU), critically ill patients receive some supportive care, including nutritional support and mobilization in order to restore or maintain organ function. In critically ill TBI patients, early nutritional support via enteral route is of particular interest due to the enhanced neurological recovery, shortened LOS, and reduced mortality (Härtl et al., 2008; Sacks et al., 1995).

Multiple lines of evidence have illustrated a direct correlation between certain dietary agents and reduced levels of inflammatory markers (Dibaba et al., 2014; Ramezani et al., 2014). More specifically, providing ICU patients with immune‐modulating nutrients (e.g., antioxidants, omega‐3 fatty acids, glutamine, and arginine) not only prevents malnutrition but also results in favorable alterations in inflammatory components, lipid profiles, antioxidant levels, intestinal microbial balance, and their acquired immune function (Calder, 2004; Hagen et al., 2009; Hegazi & Wischmeyer, 2011). However, there is a scarcity of clinical knowledge about assessing the effect of antioxidant‐rich enteral formulas on the clinical and biochemical outcomes of ICU patients. The dietary inflammatory index (DII®) is a literature‐derived tool, specifically developed to evaluate the inflammatory potential of food components on inflammatory biomarkers, such as C‐reactive protein (CRP) (Shivappa et al., 2014). To our knowledge, there is no intervention study based on the DII score in critically ill TBI patients. Given that and due to the paucity of data on the effect of the DII score on metabolic and inflammatory responses in critically ill patients, this pilot study aimed to assess the effects of a low DII score enteral formula on metabolic and inflammatory factors of TBI patients admitted to ICU.

## 2 MATERIALS AND METHODS

### 2.1 Study design

This single‐center, prospective, randomized, single‐blind, controlled clinical trial was conducted on 20 patients with TBI admitted to the neurosurgical ICU of Shahid Kamyab Hospital (Mashhad, Iran) from October 2018 to August 2019. The study was retrospectively registered in the Iranian Registry of Clinical Trials (IRCT; registry number: IRCT20180515039674N1). The study protocol (Jandari et al., 2021) was approved by the Research Ethics Committee of Mashhad University of Medical Sciences, Mashhad, Iran (reference approval number: IR.MUMS.REC.1397.149).

### 2.2 Patients and variables

Twenty TBI patients (18–65 years) with clinical manifestations indicative of poor prognosis were enrolled in this study. Data collected included the diagnosis upon ICU admission, demographic information, and medical history. Informed consent was obtained from all the patients or their legal representatives. *The inclusion criteria* were as follow: age between 18 and 65 years, moderate or severe trauma (GCS index 4–14), having normal functioning gastrointestinal tract, and indications of EN. The eligible patients were enrolled within 24–48 h after ICU admission. *The exclusion criteria* were as follows: failure to start EN within the first 48 h of admission, any history of underlying diseases (including heart disease, diabetes, congenital and immunological disorders, renal and hepatic failure, and pancreatitis), receiving multiple transfusions, enteral feeding intolerance, and less than 96 h ICU LOS. Patients with body mass index (BMI) <18.5 kg/m^2^, and patients who received total parenteral nutrition also were excluded from the study.

### 2.3 Randomization and blinding

The study group's randomization was carried out by the first author based on the inclusion and exclusion criteria. The patients were stratified upon randomization based on their age (18–65 years), gender, and disease severity based on the acute physiology and chronic health evaluation (APACHE) II score in order to ensure the equal distribution of these variables in both groups. Due to the difference in the colors of the intervention and traditional formulas in this study, the physician and nurses could not be blinded regarding patient allocation. In contrast, the patients and outcome evaluators were blinded, and the investigator was not directly involved in the intervention process and patients' care. Laboratory personnel, pathologists, and statisticians also were blinded to the treatment allocation.

### 2.4 Study measurements

Demographic data, the severity of the condition, inflammatory factors, and blood chemistry were assessed for all patients. The severity of the condition was assessed using the Acute Physiology and Chronic Health Evaluation (APACHE II) scoring system, the Sequential Organ Failure Assessment (SOFA) scoring system, and the Simplified Acute Physiology Score (SAPS) II (Zabolotskikh et al., 2012). The levels of inflammatory factors were evaluated by measuring hs‐CRP and TNF‐α using ELISA tests. Nutritional status was assessed using the nutrition risk in critically ill (NUTRIC) score and by measuring BMI and mid‐arm circumference. Lipid profiles, including triglyceride (TG), total cholesterol, high‐density lipoprotein (HDL), and low‐density lipoprotein (LDL) cholesterol, and fasting blood sugar (FBS) were also measured. Furthermore, liver function tests, including aspartate aminotransferase (AST), alanine aminotransferase (ALT), alkaline phosphatase (ALP), and total and direct bilirubin, were also assessed for each patient. Kidney function tests, including blood urea and creatinine, were also assessed.

### 2.5 Procedures

#### 2.5.1 Phase 1: Low‐DII score formula

In order to design a new formula with anti‐inflammatory properties, the Karen Pharma and Food Supplement Co. (PNC), Standard EnteraMeal formula was fortified based on the DII score of nutrients. Specific amounts of some vitamins (includes vitamins A, B_1_, B_2_, B_3_, B_5_, B_6_, B_9_, C, and D_3_), minerals (Mg, Se, and Zn), and two pharmaco‐nutrients (saffron and turmeric) with a negative inflammatory index score which have anti‐inflammatory properties were considered. Of note, the amount of these additional nutrients in the new formula was considered between the amount of the recommended daily allowance (RDA) and the upper limit (UL) of intake. In this way, not only is the patient's RDA provided via an average daily intake of 2000 cc formulation powder, but the person's inflammatory status improves without exceeding the UL. After consulting with the pharmaceutics advisory professors, a new formulation was designed and sent to James R. Hebert and Nitin Shivappa (advisory professors in the field of DII) for approval. The nutrient content of the formula is presented in Table 1. The sources and kinds of added nutrients are mentioned in Table 2.

**TABLE 1:** Nutritional content of standard enteral formula and low inflammatory index formula after the mixture.

Nutritional information	Low inflammatory index formula per 100 mL	Standard formula per 100 mL	Nutritional information	Low inflammatory index formula per 100 mL	Standard formula per 100 mL
Total DII score	−3.75	−2.69	Vitamin B12	0.53 mcg	0.53 mcg
Energy	100 Kcal	100 Kcal			
Protein	3.5 g	3.5 g	Sodium	98.6 g	98.6 mg
Carbohydrate	14.16 g	14.16 g	Potassium	142.4 g	142.4 mg
Fiber	0.44 g	0.44 g	Calcium	55 mg	55 mg
Total fat	3.5 g	3.5 g	Magnesium	27 mg	23.7 mg
Vitamin A	100 mcg	63.69 mcg	Chloride	83.26 g	83.26 mg
Vitamin E	10 mg	1.13 mg	Phosphors	52.4 mg	52.4 mg
Vitamin D_3_	1.66 mcg	0.5 mcg	Iron	0.93 mg	0.93 mg
Vitamin K1	6.53 mcg	6.53 mcg	Zinc	1.34 mg	0.92 mg
Vitamin C	66.66 mg	9.55 mg	Copper	90 mcg	90 mcg
Vitamin B_1_	0.33 mg	0.15 mg	Manganese	0.13 mg	0.13 mg
Vitamin B_2_	0.67 mg	0.18 mg	Iodine	9.53	9.53 mcg
Niacin	2 mg	1.25 mg	Chromium	1.93 mcg	1.93 mcg
Pantothenic acid	0.66 mg	0.66 mg	Fluoride	0.2 mg	0.2 mg
Vitamin B_6_	0.34 mg	0.13 mg	Selenium	13.3 mcg	3.5 mcg
Biotin	15.9 mcg	15.9 mcg	Turmeric	0.1 g	0
Folic acid	33.33 mcg	25.5 mcg	Saffron	0.02 g	0

**TABLE 2:** Source of added nutrients to standard formula.

Nutrients	Company
Retinyl acetate	Sigma
Ascorbic acid	Merck
Tocopherol acetate	Sigma
Cholecalciferol	Sigma
Thiamine hydrochloride	Sigma
Riboflavin	Sigma
Nicotinic acid	Sigma
Pyridoxine hydrochloride	Sigma
Folic acid	Sigma
Magnesium oxide	Merck
Zinc sulfate	Merck
Sodium selenite	Merck

#### 2.5.2 Phase 2: Intervention process

**FIGURE 1:**
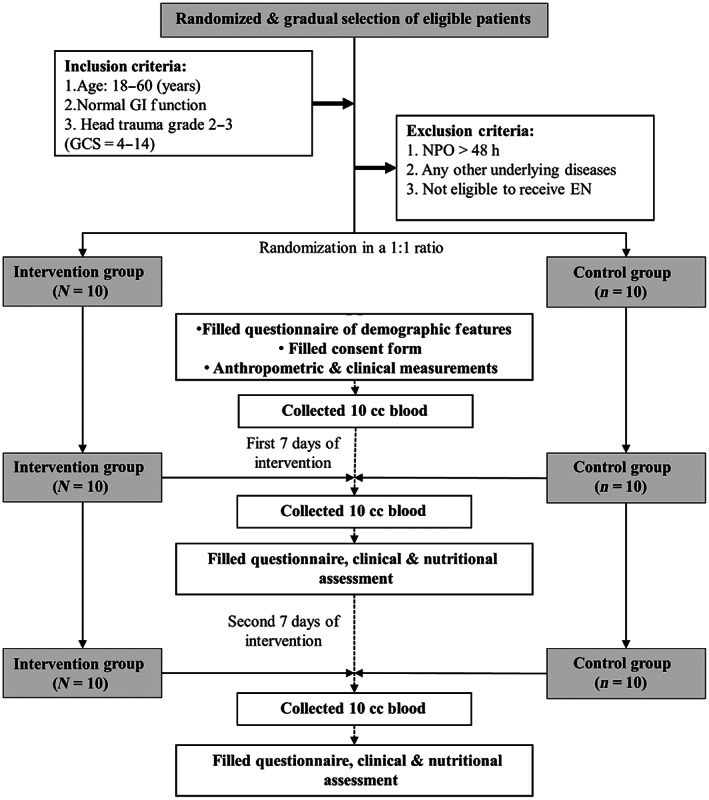
Intervention process flow chart.

The patients were selected via non‐probability purposive sampling based on the inclusion and exclusion criteria within 24–48 h after admission to the ICU randomly divided into two groups. Data were collected using observational laboratory methods. Two treatment modalities were implemented after obtaining the demographic data (age, gender, diagnosis upon admission, and classification of hospitalization). Among the other collected data were personal information of the patients, medicinal and medical history, and informed consent obtained from the patients or their legal representatives. Following that, the patients were randomly divided into two groups control and intervention. Hemodynamic resuscitation and stabilization were carried out within the first 24–48 h of admission. Afterward, EN was initiated to provide 80%–100% of the energy requirements of the patients (25 kcal/kg body weight). The amount of formula required for each patient was determined individually, and the treatment commenced within the first 24–48 h of hospitalization (seven times every 24 h). The intervention continued for 14 days. Blood sampling was performed after the last gavage meal at night, similarly in both groups on days 0, 7, and 14 (Figure 1).

### 2.6 Statistical analysis

The Statistical Package for Social Sciences (SPSS) software for Windows version 18 (IBM Inc.) was used for data analysis. Between‐group comparison of nominal or categorical variables was ascertained with the use of chi‐square test or Fisher's exact test. After determining normality using the Shapiro–Wilk test, the between‐group comparison of continuous variables was ascertained using independent sample *t*‐ or Mann–Whitney *U*‐test (non‐normal distribution). Analysis of covariance (ANCOVA) test was used to determine the mean change and effect size with the adjustment for sex and age. *p*‐value < .05 was considered statistically significant.

## 3 RESULTS

A total of 67 consecutive patients with TBI were admitted to the ICU from October 2018 to August 2019; 20 patients met all eligibility criteria and then underwent randomization and were followed until hospital discharge. Trial patients were randomly allocated to the intervention or control group at the baseline of the study, and the demographic characteristics variables of the patients were not statistically significant between the two groups. All patients received either standard or anti‐inflammatory formula for 14 days. The majority of patients were male; all patients in the intervention group and eight of the control group (*p*‐value: .47). The mean (±SD) age and weight were 36.40 ± 18.72 and 38.50 ± 16.83 years and 76.30 ± 6.06 and 73.50 ± 11.27 kg in the intervention and control groups, respectively. In each trial group, 30% and 20% of participants had subarachnoid and intracranial hemorrhage, respectively. Subdural hemorrhage was present in 30% of the control group but in only 20% of the intervention group and other participants had contusion (*p*‐value: .30). The comparison of the clinical, nutritional, and laboratory characteristics at the baseline showed no significant differences between the trial groups (Tables 3 and 4).

**TABLE 3:** Comparison of baseline clinical and nutritional characteristics between the trial groups.

Variable	Intervention group (Mean ± SD) (*N* = 10)	Control group (Mean ± SD) (*N* = 10)	*p*‐value^a^
Glasgow Coma Scale	6.20 ± 1.07	6.20 ± 1.32	.47
Oxygen saturation; %	95.62 ± 2.88	96.27 ± 4.98	.72
Pulse rate; bpm	92.90 ± 16.68	89.80 ± 15.84	.67
MAP; mmHg	89.80 ± 8.06	97.7 ± 9.36	.06
APACHE II	13.90 ± 2.42	14.00 ± 3.43	.94
SOFA	6.30 ± 1.57	6.30 ± 2.71	.99
SAPS II	39.10 ± 8.28	36.30 ± 11.86	.55
NUTRIC score II	3.50 ± 0.97	3.40 ± 1.26	.84
MAC; cm	27.60 ± 2.45	27.50 ± 3.92	.95
Energy expenditure; Kcal	1925.00 ± 272.08	1920.00 ± 278.09	.97

*Note*: Data are presented as mean ± standard deviation.

Abbreviations: APACHE, acute physiology and chronic health evaluation; bpm, beats per minute; GCS, Glasgow Coma Scale; MAC, mid‐arm circumference; MAP, mean arterial pressure; NUTRIC, nutrition risk in critically ill; SAPS, simplified acute physiology score; SOFA, sequential organ failure assessment.

^a^ *p*‐values were obtained from an independent sample *t*‐test.

**TABLE 4:** Comparison of baseline laboratory parameters between the trial groups.

Variable	Intervention group (Mean ± SD) (*N* = 10)	Control group (Mean ± SD) (*N* = 10)	*p*‐value^a^
Albumin; g/dL	3.7 ± 0.43	3.6 ± 0.43	.61
Total protein; g/dL	5.97 ± 0.84	5.87 ± 0.49	.75
Albumin/total protein	0.62 ± 0.060	0.61 ± 0.064	.72
TNF‐α	5.81 ± 2.38	5.96 ± 2.87	.90
hs‐CRP; mg/dL	18.15 ± 1.29	17.58 ± 1.32	.34
Fasting blood sugar; mg/dL	136.9 ± 35.40	145.5 ± 32.63	.58
Total cholesterol; mg/dL	112.1 ± 36.60	128.60 ± 42.74	.36
LDL‐c; mg/dL	70.60 ± 32.85	69.30 ± 34.18	.93
HDL‐c; mg/dL	32.90 ± 12.45	35.4 ± 3.62	.55
Triglyceride; mg/dL	105.5 [91.5–119.6]	92.5 [82.8–136.4]	.88^b^
AST; IU/L	67.5 [38–103.2]	88 [54.1–110]	.82^b^
ALT; IU/L	37.5 [29.6–65.9]	39.5 [32.6–52.9]	.91^b^
ALP; IU/L	157 [143.2–199.4]	157 [150–164.6]	.99^b^
D‐Bil; mg/dL	0.305 [0.278–0.485]	0.275 [0.233–0.320]	.71^b^
T‐Bil; mg/dL	0.880 [0.800–1.045]	1.165 [1.000–1.300]	.08^b^
BUN; mg/dL	35.7 ± 8.33	39.1 ± 13.19	.49
Creatinine; mg/dL	0.962 ± 0.204	0.974 ± 0.216	.90
WBC (×1000/μL)	16.93 ± 9.16	11.84 ± 4.58	.13
PLT (×1000/μL)	182 ± 61	178 ± 55	.86

*Note*: Data are presented as mean ± standard deviation or median [interquartile range].

Abbreviations: ALP, alkaline phosphatase; ALT, alanine aminotransferase; AST, aspartate aminotransferase; BUN, blood urea nitrogen; D‐Bil, direct bilirubin; HDL‐c, high‐density lipoprotein cholesterol; hs‐CRP, high‐sensitivity C‐reactive protein; LDL‐c, low‐density lipoprotein cholesterol; T‐Bil, total bilirubin; TNF‐α, tumor necrosis factor‐alpha.

^a^ *p*‐values were obtained from independent sample *t*‐test unless otherwise indicated.

^b^ *p*‐values were obtained from Mann–Whitney *U*‐test.

The mean total calorie intake on days 7 and 14 were 1185 ± 234 and 1333 ± 213 Kcal in the intervention and 1231 ± 226 and 1365 ± 151 Kcal in the control groups, respectively. The trial groups did not differ significantly in terms of energy intake. Adjusted mean changes (95% CI) in laboratory characteristics from baseline to 7‐ and 14‐day intervention are summarized in Table 5. Serum TC concentration and PLT count increased significantly, whereas serum hs‐CRP and creatinine concentrations and WBC count decreased significantly in the intervention group during the first week of the intervention. The serum BUN, LDL‐c, and TC concentrations and PLT count during the first week of the intervention increased significantly in the control group. Other parameters did not significantly change in either group during this period. The two groups did not differ significantly with respect to laboratory parameters within the first week of intervention. The serum hs‐CRP and FBS concentrations and PLT and WBC count in the intervention group and the serum TNF‐α, FBS, LDL‐c, and creatinine concentrations and PLT count in the control group changed significantly from baseline to 14‐day intervention. Other parameters did not significantly change in either group during this period. Among these variables, only hs‐CRP, FBS, and LDL‐c showed a significant reduction in the intervention group compared with the control group.

**TABLE 5:** Adjusted mean change in biochemical factors from baseline to 1‐ and 2‐week follow‐up.

Variables	Short‐term change			Long‐term change		
	Intervention (*N* = 10)	Control (*N* = 10)	Effect size	Intervention (*N* = 10)	Control (*N* = 10)	Effect size
TNF‐α	0.78 (−0.86, 2.42)	1.02 (−0.62, 2.66)	−0.23 (−2.63, 2.16)	1.10 (−0.91, 3.11)	2.03 (0.03, 4.04)*	−0.93 (−3.86, 1.99)
hs‐CRP; mg/dL	−1.39 (−2.47, −0.32)*	−0.13 (−1.21, 0.94)	−1.26 (−2.83, 0.30)	−2.73 (−3.67, −1.79)*	0.65 (−0.29, 1.58)	−3.37 (−4.73, −2.01)*
Alb; g/dL	0.01 (−0.15, 0.16)	0.04 (−0.11, 0.19)	‐ 0.03 (−0.26, 0.19)	−0.03 (−0.22, 0.17)	−0.09 (−0.29, 0.10)	0.07 (−0.22, 0.35)
TP; g/dL	0.19 (−0.30, 0.68)	0.21 (−0.28, 0.70)	−0.02 (−0.74, 0.69)	0.33 (−0.27, 0.92)	0.17 (−0.43, 0.77)	0.16 (−0.71, 1.03)
Alb:TP ratio	−0.022 (−0.07, 0.02)	−0.013 (−0.06, 0.03)	−0.01 (−0.08, 0.06)	−0.04 (−0.09, 0.01)	−0.03 (−0.08, 0.02)	0.01 (−0.09, 0.07)
FBS; mg/dL	−13.8 (−58.63, 30.91)	−21.3 (−66.01, 23.51)	7.5 (−57.70, 72.52)	−41.7 (−51.74, −31.6)*	−22.9 (−32.61, −13.3)*	−18.7 (−32.86, −4.64)*
TC; mg/dL	26.51 (5.64, 47.57)*	59.7 (38.80, 80.63)*	−33.2 (−60.6, 0.87)	16.8 (−2.62, 36.30)	42.9 (23.51, 62.44)	−26.2 (−54.43, 2.14)
LDL‐c; mg/dL	10.1 (−11.42, 31.62)	36.9 (16.18, 57.63)*	−26.8 (−57.05, 3.43)	2.1 (−16.08, 20.29)	35.8 (17.60, 53.98)*	−33.7 (−60.16, −7.21)*
HDL‐c; mg/dL	0.24 (−6.83, 7.30)	1.76 (−5.31, 8.83)	−1.52 (−11.81, 8.76)	−1.78 (−8.52, 4.96)	0.88 (−5.86, 7.63)	−2.67 (−12.48, 7.14)
TG; mg/dL	52.1 (−25.78, 129.99)	55.4 (−22.49, 133.28)	−3.3 (−116.6, 110.1)	43.4 (−33.54, 120.32)	32.61 (−44.32, 109.5)	10.78 (−101.2, 122.7)
AST; IU/L	−32 (−144.75, 80.73)	49.8 (−62.94, 162.56)	−81.82 (−245.9, 82.2)	−64.7 (−143.52, 13.95)	20.18 (−58.55, 98.92)	−84.9 (−199.5, 29.62)
ALT; IU/L	47.5 (−89.1, 184.13)	55.3 (−81.25, 191.94)	−7.81 (−206.6, 191)	54.2 (−39.44, 147.9)	37.6 (−55.98, 131.32)	16.54 (−119.7, 152.8)
ALP; IU/L	54.2 (−39.44, 147.9)	37.7 (−55.9, 131.3)	16.5 (−119.7, 152.8)	144.9 (32.5, 257.4)	280.8 (168.4, 393.3)	−135.9 (−299.5, 27.7)
D‐Bil; mg/dL	0.015 (−1.10, 1.13)	−0.88 (−1.99, 0.23)	0.89 (−0.72, 2.52)	0.032 (−1.21, 1.27)	−1.01 (−2.25, 0.23)	1.04 (−0.76, 2.85)
T‐Bil; mg/dL	−0.053 (−1.41, 1.31)	−1.06 (−2.42, 0.29)	1.01 (−0.97, 2.99)	−0.084 (−1.49, 1.32)	−1.18 (−2.58, 0.22)	1.10 (−0.94, 3.14)
BUN; mg/dL	−0.69 (−7.87, 6.48)	7.79 (0.62, 14.97)*	−8.49 (−18.94, 1.94)	1.02 (−4.83, 6.87)	1.37 (−4.47, 7.23)	−0.35 (−8.87, 8.16)
Creatinine; mg/dL	−0.15 (−0.29, −0.02)*	−0.09 (−0.23, 0.05)	−0.06 (−0.26, 0.14)	−0.11 (−0.26, 0.05)	−0.19 (−0.35, −0.04)*	0.089 (−0.14, 0.32)
WBC; (×1000/μl)	−4.91 (−8.78, −1.07)*	0.13 (−3.71, 3.97)	−5.04 (−10.62, 0.55)	−5.79 (−10.35, −1.23)*	−1.44 (−6,01, 3.11)	−4.35 (−10.99, 2.28)
PLT; (×1000/μl)	102.8 (31.26, 174.3)*	91.68 (20.14, 163.2)*	11.13 (−92.99, 115.2)	207.6 (78.15, 337.1)*	219.8 (90.3, 349.2)*	−12.1 (−200.5, 176.2)

*Note*: Data are presented as mean (95% confidence interval). Values were obtained from ANCOVA test with the adjustment for sex and age.

Abbreviations: ALP, alkaline phosphatase; ALT, alanine aminotransferase; AST, aspartate aminotransferase; BUN, blood urea nitrogen; D‐Bil, direct bilirubin; FBS, fasting blood sugar; HDL‐c, high‐density lipoprotein cholesterol; hs‐CRP, high‐sensitivity C‐reactive protein; LDL‐c, low‐density lipoprotein cholesterol; T‐Bil, total bilirubin; TC, total cholesterol; TG, triglyceride; TNF, tumor necrosis factor.

* *p*‐value < .05.

**FIGURE 2:**
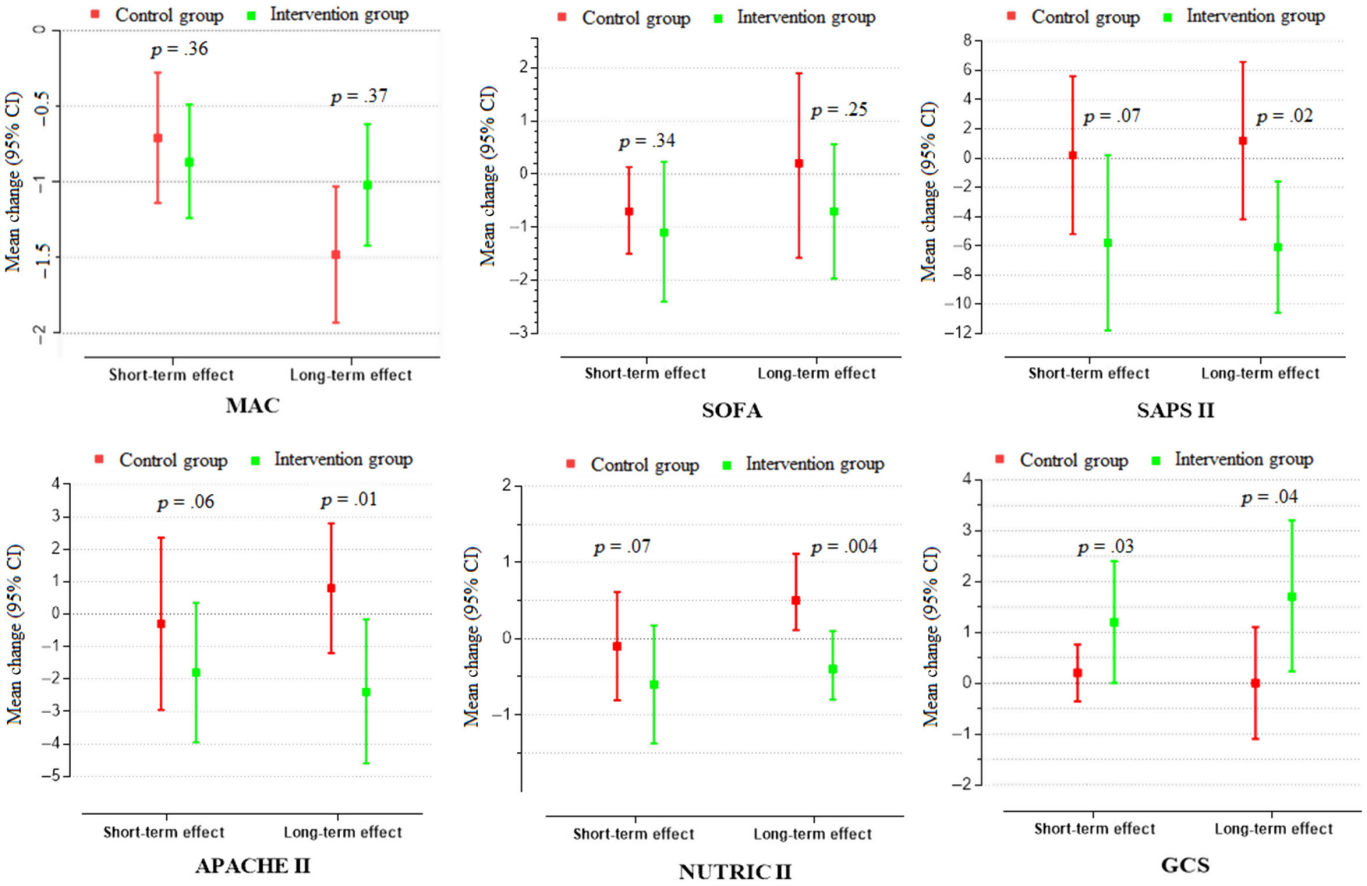
Age‐ and sex‐adjusted mean change for biochemical factors in the intervention group (*N* = 10) and control group (*N* = 10) from baseline to 1‐week (short‐term) and 2‐week (long‐term) follow‐ups. APACHE, acute physiology and chronic health evaluation; GCS, Glasgow Coma Scale; MAC, mid‐arm circumference; NUTRIC score II, Nutrition risk in critically ill score II; SAPS, simplified acute physiology; SOFA, sequential organ failure assessment.

**FIGURE 3:**
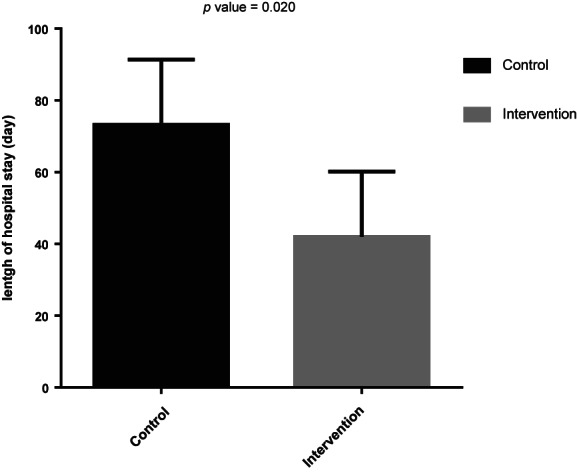
Comparison of length of hospital stay between the intervention group (*N* = 10) and control group (*N* = 10). Values were obtained from the analysis of covariance (ANCOVA) test with the adjustment for sex and age.

Multivariable‐adjusted mean changes (95% CI) in clinical characteristics are summarized in Figure 2. Within‐group comparisons showed that only MAC decreased significantly in both groups, and GCS scores increased significantly in the intervention group during the first 7 days of the intervention. The between‐group comparison demonstrated that only the average increase in GCS score was significantly higher in the intervention group compared to the control group during this period (*p*‐value = .032). The mean of APACHE II and SAPS II scores and MAC during the 14 days of the intervention decreased and GCS increased significantly in the intervention group. On the other hand, in the control group, MAC decreased and NUTRIC scores increased significantly. The comparison of the changes in clinical parameters between the study groups following 14 days of intervention demonstrated that the average change in GCS score was significantly higher and also change in APACHE II, SAPS II, and NUTRIC scores were significantly lower in the intervention group compared with the control group. Moreover, the adjusted mean (95% CI) for hospital length of stay (LOS) was 73.21 (55, 91.4) days for the control group and 41.99 (23.80, 60.18) days for the intervention group (Figure 3).

## 4 DISCUSSION

The overall results indicated that designing an enteral formula with a low‐DII score for critically ill patients is associated with a significantly greater reduction in serum hs‐CRP, LDL‐c, and FBS levels compared to the group receiving standard formula. On the other hand, the average change in GCS score was significantly higher, and changes in APACHE II, SAPS II, and NUTRIC scores were significantly lower in the intervention group compared to the control group. Moreover, the intervention group had a significantly shorter LOS compared to the control group.

Our results demonstrated that although hs‐CRP levels did not differ significantly between the two groups on day 7, it was statistically significant on day 14. Additionally, the hs‐CRP decrease trend was statistically significant in the intervention group, while no significant changes were observed in the control group. This is in line with previous findings, demonstrating a significant positive relationship between DII and several inflammatory biomarkers, including IL‐6 (Shivappa et al., 2014, 2015) and hs‐CRP concentration (Shivappa et al., 2014). CRP is a non‐specific biomarker reflecting any inflammatory condition that commonly increases 4–6 h after trauma or surgery (Vincent et al., 2011). The declining trend in hs‐CRP levels indicates the patient's entry into the anabolic phase; at this point, nutritional intervention can be effective (Shivappa et al., 2014). The hs‐CRP test is widely used to accurately measure low levels of CRP and diagnose vascular inflammation (Ridker et al., 1998). In a Lothian birth cohort study, Corley et al. (2019) investigated the association between the energy‐adjusted DII score (derived from food frequency questionnaires) and the serum level of hs‐CRP. Their result indicated that DII/E‐DII, as a valid index, has the ability to estimate inflammatory markers, and diet seems to play an important role in regulating the inflammatory conditions in the body (Corley et al., 2019). On closer inspection, inflammatory biomarkers such as TNF‐a and IL‐1 have several common pro‐inflammatory properties, such as prostaglandin E2 (PGE2) production as well as the activation of collagenase (Feghali & Wright, 1997), which in cases such as rheumatoid arthritis can cause joint damage. Vitamins and minerals can affect cyclooxygenase and lipoxygenase pathways, modulate the production of prostaglandins such as PGE2, and thereby alter the response to injuries and infections (Mahan & Raymond, 2016).

Recently, this effect has been studied especially in patients with malnutrition and it was observed that diets rich in n‐3 fatty acids, arginine, glutamine, and vitamins C and E improve clinical outcomes including infections, inflammation, and complications that occur in patients after surgery or trauma (Klek et al., 2011; Marano et al., 2013). In accordance with previous literature, our enriched low‐DII formula with anti‐inflammatory properties may affect this process in critically ill patients by increasing the intake of dietary antioxidants.

The present study also suggested that LDL‐c levels in the group receiving low‐DII score formula decrease significantly compared to the group receiving standard formula. Previous studies have proposed the mechanisms underlying inflammation‐related alterations in lipid metabolism, leading to increased levels of LDL‐c, lipoprotein (a), and triglycerides and decreased HDL levels (Khovidhunkit et al., 2004). A recent study conducted by Phillips et al. (2018) found that diets with higher DII scores (more pro‐inflammatory diets) caused unfavorable alterations in lipid profile, including elevated LDL‐c levels. Moreover, Ridker et al. (1998) found that higher levels of hs‐CRP are significantly associated with items of metabolic syndrome including hypertriglyceridemia, low HDL level, obesity, hypertension, and abnormal glucose metabolism. These findings are consistent with another study conducted by Vahid et al. (2017) on 400 patients using a food frequency questionnaire. In that study, they determined the inflammatory index of individuals' diet and found that subjects in the third tertile of DII had significantly higher levels of FBS, HbA1C, LDL, TG, and body fat but lower levels of HDL. Their results also showed that those who consumed a more pro‐inflammatory diet were at higher risk for prediabetes (Vahid et al., 2017). Decades ago, scientists found that inflammation exacerbates insulin resistance, which can eventually lead to higher FBS levels (Wellen & Hotamisligil, 2005). Hyperglycemia is probably a sign of an active inflammatory response that is accompanied by an increase in tumor necrosis factor (TNF) activated by nuclear factor kappa b (NF‐β). Hyperglycemia can also increase the production of reactive oxygen or nitrogen species and counteracts insulin activity in the human body (Langouche et al., 2005). This pathway explains the significant decrease in FBS levels in the intervention group compared to the control group in our study.

Regarding clinical parameters, the GCS score, as a scoring system for assessing the TBI severity, was significantly higher in the intervention group than in the control group. Previous studies have shown that the lower the score of GCS in TBI patients, the higher the unfavorable outcomes following trauma and mortality tend to be (Gilani et al., 2017; McIntyre et al., 2013). Traumatic brain injury may cause severe brain inflammation, resulting in cerebral edema and elevated intracranial pressure. This condition can worsen the GCS score (Strauss, 2008); therefore, reducing inflammation can help to maintain GCS. Inflammation in critically ill patients is characterized by major alterations in energy, macronutrient, and micronutrient requirements, as well as changes in metabolism and reduced nutrient absorption. These destructive processes ultimately increase the risk of malnutrition in critically ill patients admitted to the ICU (Ndahimana & Kim, 2018). The NUTRIC score, a new screening tool assessing patients' nutritional risk (Heyland et al., 2011), was found to be significantly higher in the group that received low DII formula than the control group on day 14. The three prognostic indices, APACHE II, SOFA, and SAPS II scores, are highly regarded in clinical assessments due to the use of multiple physiological variables (Lewandowski & Lewandowski, 2003). According to our results, the APACHE II score did not differ significantly between the intervention and control groups at baseline and day 7. On day 14, however, this score was significantly lower in the intervention group compared to the control group. On the other hand, the trend of the SAPS II scores in the intervention group was significantly downward throughout the long‐term intervention period, while this trend was upward in the control group. Moreover, a significant difference was found between the two groups. In a retrospective cohort study, Basil et al. examined the prognostic indices (APACHE II, SOFA, and SAPS 3) as well as biological markers (hs‐CRP/Albumin and lactate) in 765 patients to predict the mortality rate of surgical patients admitted to the ICU. They found out that prognostic indices such as APACHE II, APACHE DP (APACHE death probability), SAPS 3, and SAPS 3 DP (SAPS 3 death probability) have greater predictability than biological markers such as lactate, albumin, CRP, and CRP/albumin in patients' mortality admitted to the ICU (Basile‐Filho et al., 2019).

Mean hospital LOS in the intervention group was significantly lower than the control group. This is consistent with our other findings on reducing the level of inflammation and the severity of the disease in the ICU. Similarly, a study of 193 patients who underwent colorectal surgery found that lower hs‐CRP levels on the second postoperative day (POD2) were associated with a shorter hospital LOS (Krpata et al., 2013).

In the present study, a homogeneous group of TBI patients was enrolled, which adjusted the impact of confounding variables in both study groups. Patients were carefully and regularly monitored by recording nutritional intake and clinical testing, a major advantage over more poorly controlled studies. In the present pilot study, although the sample size was calculated based on the study of Lee et al. (2016), a larger sample size would have increased precision. In this study, the samples were obtained from a single hospital. With these encouraging results as background, future multicenter studies should be planned to obtain more robust results. Moreover, it might be advisable to extend follow‐up for longer periods of time.

## 5 CONCLUSIONS

This study demonstrated that low‐DII score formula (which contains vitamins, salts, and nutrients with antioxidant properties) may improve inflammatory factors (hs‐CRP), metabolic biomarkers, and clinical outcomes in critically ill TBI patients admitted to the intensive care unit. However, despite the important clinical results of the present study, it seems that the formula used in this study needs to be evaluated in larger patient groups and multicenter trials in order to produce more robust and generalizable results that can help guide clinical practice.

## AUTHOR CONTRIBUTIONS

SJ, RR, MB, and MS contributed to the conception or design. SJ, NM, and DS drafted the manuscript. SJ, RR, NS, JRH, and AK prepared the low‐DII formula. NS and JRH also calculated the formula DII. SJ and SY collected samples at ICU while DS and SZM contributed to acquisition, analysis, or interpretation. RR, MB, LJ, and MS critically revised the manuscript and RR, LJ, and MS gave final approval. MS also agrees to be accountable for all aspects of work ensuring integrity and accuracy.

## FUNDING INFORMATION

This study was supported by a grant from the Mashhad University of Medical Sciences.

## CONFLICT OF INTEREST STATEMENT

Dr. James R. Hébert owns a controlling interest in Connecting Health Innovations LLC (CHI), a company that has licensed the right to his invention of the dietary inflammatory index (DII®) from the University of South Carolina in order to develop computer and smartphone applications for patient counseling and dietary intervention in clinical settings. Dr. Nitin Shivappa is an employee of CHI. The subject matter of this paper will not have any direct bearing on that work or has that activity exerted any influence on this project.

## Data Availability

The data could be available at the request of the editor.
